# Validation of the i-STAT and HemoCue systems for the analysis of blood parameters in the bar-headed goose, *Anser indicus*

**DOI:** 10.1093/conphys/cov021

**Published:** 2015-05-19

**Authors:** T. S. Harter, M. Reichert, C. J. Brauner, W. K. Milsom

**Affiliations:** Department of Zoology, University of British Columbia, 6270 University Boulevard, Vancouver, BC, CanadaV6T 1Z4

**Keywords:** Bird, carbon dioxide, oxygen, pH, portable clinical analyser

## Abstract

Every year, bar-headed geese (*Anser indicus*) perform some of the most remarkable trans-Himalayan migrations, and researchers are increasingly interested in understanding the physiology underlying their high-altitude flight performance. A major challenge is generating reliable measurements of blood parameters on wild birds in the field, where established analytical techniques are often not available. Therefore, we validated two commonly used portable clinical analysers (PCAs), the i-STAT and the HemoCue systems, for the analysis of blood parameters in bar-headed geese. The pH, partial pressures of O_2_ and CO_2_ (*P*O_2_ and *P*CO_2_), haemoglobin O_2_ saturation (sO_2_), haematocrit (Hct) and haemoglobin concentration [Hb] were simultaneously measured with the two PCA systems (i-STAT for all parameters; HemoCue for [Hb]) and with conventional laboratory techniques over a physiological range of *P*O_2_, *P*CO_2_ and Hct. Our results indicate that the i-STAT system can generate reliable values on bar-headed goose whole blood pH, *P*O_2_, *P*CO_2_ and Hct, but we recommend correcting the obtained values using the linear equations determined here for higher accuracy. The i-STAT is probably not able to produce meaningful measurements of sO_2_ and [Hb] over a range of physiologically relevant environmental conditions. However, we can recommend the use of the HemoCue to measure [Hb] in the bar-headed goose, if results are corrected. We emphasize that the equations that we provide to correct PCA results are applicable only to bar-headed goose whole blood under the conditions that we tested. We encourage researchers to validate i-STAT or HemoCue results thoroughly for their specific study conditions and species in order to yield accurate results.

## Abstract (precis)

Our results indicate that the i-STAT and HemoCue systems can be useful tools to measure many (but not all) blood-gas and acid-base variables in the bar-headed goose. The accuracy of generated results can be improved by using the correction equations provided here, although extrapolation beyond the tested conditions should be avoided.

## Introduction

The accurate measurement of blood gases and pH in field studies on animal physiology is not easily accomplished. While proven and established methods are available in the laboratory, these techniques are typically not portable or suited for operation under most field conditions. Consequently, researchers have adopted portable clinical analysers (PCAs), such as the i-STAT system^®^ (Abbot Point of Care Inc., Princeton, NJ, USA), for the analysis of blood parameters on a broad range of animal species, often without previous validation (Stoot *et al.*, 2014). To our knowledge, no previous study has validated the i-STAT for the analysis of blood parameters in an avian species. We have previously validated the i-STAT for its use on a teleost (Harter *et al.*, 2014) and an elasmobranch species (Harter *et al.*, 2015). Results indicated that under the tested conditions and for the two species studied, the i-STAT did not report accurate values of blood parameters (except for blood pH). Therefore, and despite the lack of suitable alternatives, the i-STAT system should not be used on fish without additional validation for the particular conditions and species in question. Other researchers have validated the i-STAT system in reptiles and mammals, which are physiologically more similar to birds, compared to fish. McCain *et al.* (2010) validated the i-STAT system for the parameters Cl^−^, glucose, K^+^ and Na^+^ in a variety of reptile species; however, the reference technique was another automated human blood analyser (Hitachi 911). Wolf *et al.* (2008) validated the i-STAT for haematocrit (Hct; among other analytes) in various sea turtle species and found significantly lower Hct values in the i-STAT compared with measurements in capillary tubes. Hopper and Cray (2007) validated the i-STAT system for Hct and haemoglobin (Hb) concentration (among other analytes) in cynomolgus macaques and found the i-STAT to overestimate both of these parameters. Similar results were obtained by Larsen *et al.* (2002), where the i-STAT overestimated Hct in elephant seals, compared with an automated cell counter.

The i-STAT system was developed for the analysis of human blood; therefore, analysis is performed at 37°C (in a heated cartridge) and results are calculated assuming human blood characteristics. Based on our previous results on poikilothermic fish, measurements of blood parameters were inaccurate (Harter *et al.*, 2014, 2015) due to: (i) differences in body temperature between fish and humans and the effects of a closed system temperature increase during analysis (Malte *et al.*, 2014); (ii) the low partial pressures of CO_2_ (*P*CO_2_) in water breathers, which were outside the reportable range in the i-STAT; (iii) differences in O_2_-binding properties between human and fish Hb; and (iv) the nucleated state of red blood cells (RBCs) in fish compared with non-nucleated cells in humans. While birds also have nucleated RBCs and Hb isoforms with O_2_-binding characteristics that may differ from those found in humans, birds are homeotherms (∼41°C in bar-headed geese) and air breathers with relatively high *P*CO_2_, just like humans. Therefore, some of the major constraints for implementing the i-STAT system on fishes are likely to be absent in birds, which may allow for more accurate measurements of blood parameters.

Bar-headed geese (*Anser indicus*) perform bi-annual migrations, in which they ascend the face of the Himalayas in less than a day, climbing to 6000 m, in what has been described as ‘the highest and most iconic trans-mountain migration in the world’ (Hawkes *et al.*, 2011). Not surprisingly, there has been increasing interest among researchers in understanding the physiological adaptations that allow for these exceptional flight performances (Rollema and Bauer, 1979; Faraci, 1991; Scott and Milsom, 2006; Hawkes *et al.*, 2011; Bishop *et al.*, 2015). For ongoing and future studies, it is pivotal to generate accurate measurements of blood gases, acid–base status, haemoglobin concentration [Hb] and Hct of wild bar-headed geese under field conditions. The aim of the present study, therefore, was to validate the i-STAT system for the analysis of these blood parameters in the bar-headed goose, over a predetermined physiological range of *P*O_2_ and *P*CO_2_ and taking advantage of the naturally occurring inter-individual variability in Hct. In addition, we validated a second PCA, the HemoCue^®^ (HemoCue AB, Ängelholm, Sweden) for the analysis of [Hb] in bar-headed goose whole blood. Our goal was to identify those parameters that can be measured reliably using the i-STAT and HemoCue systems and, if applicable, provide appropriate correction equations to increase the accuracy of the data produced. We recognize that the i-STAT system can be a powerful tool for measuring blood parameters in the field, if other methods are unavailable, and may greatly contribute to the progress in a given field of research, provided accurate results are obtained. Our results should help researchers to identify the limitations of the i-STAT system and allow them to make an informed decision on whether the i-STAT is the right tool to answer their specific research questions.

## Materials and methods

### Animals and housing

Bar-headed geese (*Anser indicus*, Latham 1790) were obtained from Sylvan Heights Waterfowl Park (Scotland Neck, NC, USA) and held at the University of British Columbia animal care facilities for several months before experiments. Twelve animals (2.77 ± 0.18 kg; mean ± SD) were kept in an outside enclosure with free access to shelter, standing water and commercial waterfowl feed. Husbandry conditions and all procedures were approved by and strictly according to the guidelines specified by the Canadian Council on Animal Care (UBC protocol no. A12-0013).

### Blood collection

Each experimental day, six unanaesthetized birds were restrained by a technician, and 4 ml of blood was collected from the medial metatarsal vein into a heparinized syringe. Samples were then transferred to glass vials and stored on ice until experiments (typically < 1 h). Aliquots of blood (3 ml) were loaded into each of six Eschweiler tonometers (5 ml total volume), and Hct was measured in triplicate on 30 μl subsamples. Tonometers were incubated in a water bath, thermostated at 41°C, and were continuously flushed with a water-saturated custom-mixed gas (O_2_, CO_2_ and N_2_) from a DIGAMIX 275 6KM 422 Woesthoff pump (Bochum, Germany). Blood samples in tonometers were equilibrated with the respective gas tensions for 1 h before subsamples were taken for analysis.

### Experimental design

The 3 ml blood samples in each tonometer were sequentially equilibrated to 40, 80 and 120 mmHg *P*O_2_ by changing the composition of the gas mixture; sampling was performed after each equilibration step. This protocol was repeated on three separate days, in which *P*CO_2_ was set to 2 (∼15 mmHg), 4 (∼30 mmHg) or 6% (∼45 mmHg). Six replicate samples (a single tonometer containing blood from a single individual; *n* = 6) were run for each combination of factors, resulting in 54 samples overall (18 samples/day). To obtain 18 blood samples from 12 donor birds, six birds were sampled twice, allowing for 2 weeks of recovery between sampling points (there were no significant differences in mean Hct between the first and the second sampling).

### Sampling and analysis protocols

After equilibration, the six tonometers were sampled sequentially using heparinized, gas-tight Hamilton syringes. A subsample of 90 μl was immediately loaded into an i-STAT cartridge. Measurements were performed using the VetScan i-STAT 1 System (SN:704534-C; software version JAMS 137A/CLEW A28; Abaxis, Union City, CA, USA) with the i-STAT CG8+ cartridge test. Cartridges were stored in their original packaging at 4°C in the dark, and allowed to equilibrate to room temperature overnight prior to experiments. In addition, ∼10 μl of blood were loaded into a HemoCue 201^+^ microcuvette (HemoCue AB, Ängelholm, Sweden) for the analysis of [Hb].

Control measurements of blood parameters were carried out using established laboratory techniques. Hct was measured in triplicate using microhaematocrit capillary tubes (10 μl) and, after centrifuging at 17 000***g*** for 3 min, [Hb] was measured in triplicate with a Shimadzu UV-1800 spectrophotometer (Kyoto, Japan) using the cyanomethaemoglobin method. The [Hb] was calculated based on absorption measurements at 540 nm and using an extinction coefficient of 11. Whole blood pH, *P*O_2_ and *P*CO_2_ measurements were performed using a Radiometer BMS 3 Mk2 system, thermostated at 41°C, with Radiometer acid–base analysers PHM 71 and PHM 84 (Copenhagen, Denmark) and a Cameron Instruments OM200 (Port Aransas, TX, USA). Whole blood total O_2_ content (*T*O_2_) was measured according to Tucker (1967). The Hb-O_2_ saturation (sO_2_) was calculated from *T*O_2_ after subtracting physically dissolved O_2_ according to Boutilier *et al*. (1984) and dividing by the theoretical maximal carrying capacity of the rinsed RBCs based upon the tetrameric [Hb] obtained spectrophotometrically.

### Data analysis

All data were analysed with RStudio 0.98.1049 (RStudio Inc., Boston, MA, USA). Given that i-STAT and HemoCue report [Hb] in different units, we converted both results into millimolar using the molecular weight of human Hb. All i-STAT and HemoCue values were compared with control measurements by regression analysis using the raw data (*n* = 54). The measurement errors for the i-STAT and HemoCue values relative to control measurements were calculated as follows: δ = (PCA − control)/control × 100. The δ data were then compared with control measurements by regression analysis (*n* = 54). Normality of distribution was tested with the Shapiro–Wilk test (*P* < 0.05), and homogeneity of variances was tested with Levene's test (*P* < 0.05). The effects of *P*O_2_ and *P*CO_2_ on δ were tested on the squared values of δ (i.e. all values were positive). In some cases, this transformation led to a significant deviation of the distribution from normality, which could not be remediated by data transformation. If such was the case, the effects of *P*O_2_ and *P*CO_2_ on δ were tested with the Wilcoxon rank sum test (*P* < 0.05, *n* = 18); otherwise one-way ANOVAs were used (*P* < 0.05, *n* = 18). All data are presented as means ± SEM.

## Results

### pH

**Figure 1:**
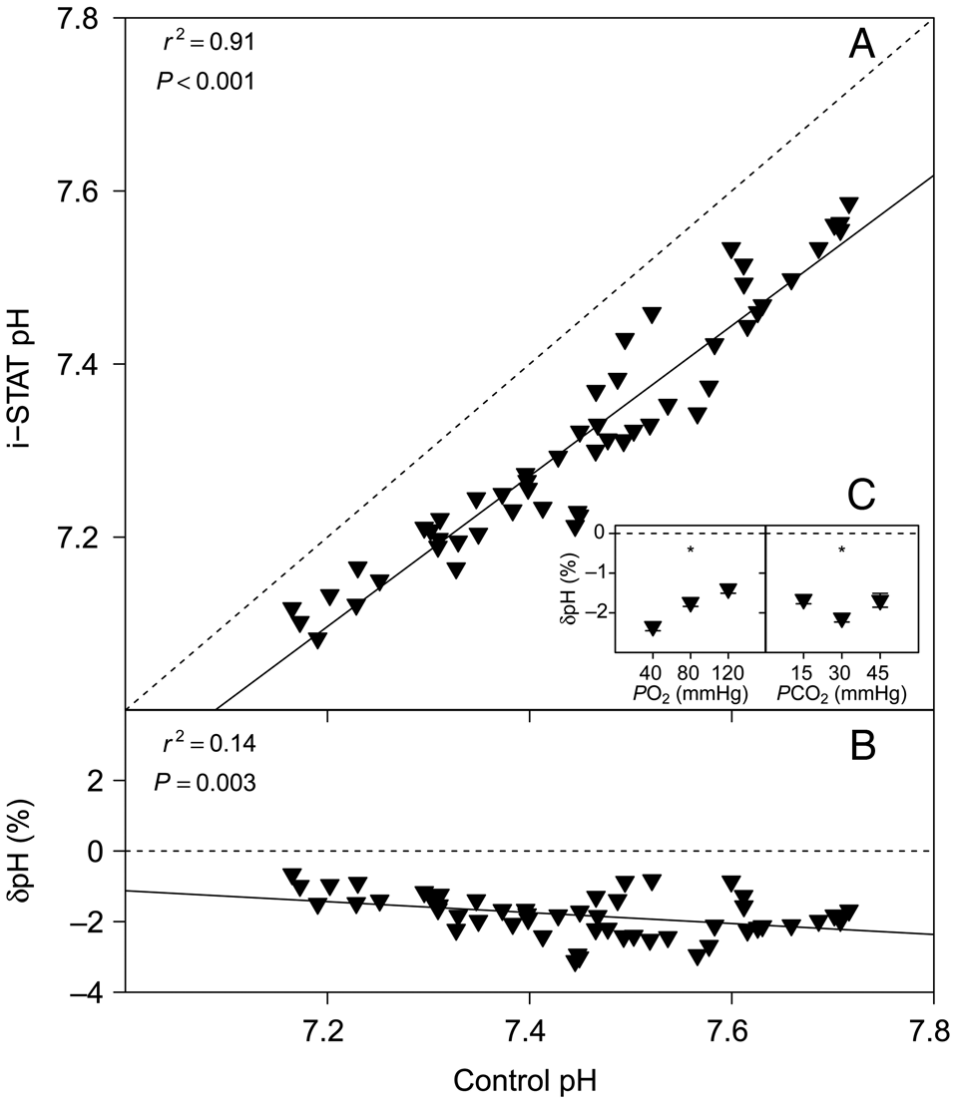
(**A**) Bar-headed goose whole blood pH measured with the i-STAT system (temperature-corrected values) vs. pH measured using a thermostated electrode (control). (**B**) The relative error of i-STAT pH measurements, δpH [in %; calculated as: (i-STAT pH − control pH)/control pH × 100] vs. control pH. Continuous lines represent the fitted linear models (see Table 1 for parameter estimates), and dashed lines represent the lines of identity. (**C**) Effects of partial pressures of O_2_ and CO_2_ (*P*O_2_ and *P*CO_2_; in mmHg) on δpH. Significant effects within treatments are indicated as * at *P* < 0.05 or NS for non-significant. Data are means ± SEM, and statistical analysis was performed on the absolute δpH values.

There was a significant linear relationship between pH measurements performed with the i-STAT and control pH measurements with a thermostated electrode (Fig. 1A). The measurement error of i-STAT pH measurements, δpH (expressed as a percentage), relative to control pH is shown in Fig. 1B and is best described by a significant linear relationship, with the equations given in Table 1. There were significant effects of *P*O_2_ (*P* < 0.001) and *P*CO_2_ (*P* = 0.014) on δpH (Fig. 1C).

**Table 1:** Parameter estimates (means ± SEM), *r*^2^ and *P*-values for the relationships between i-STAT (or HemoCue) vs. control measurements, and between i-STAT (or HemoCue) measurement errors, δ(*x*) (in %), vs. control measurements (*n* = 54)

Measurement	*a*	*b*	*c*	*r*^2^	*P*-value
pH	0.839 ± 0.281	0.869 ± 0.038		0.91	<0.001
δpH	9.703 ± 3.768	−1.547 ± 0.506		0.14	0.004
*P*O_2_	−2.152 ± 3.495	0.732 ± 0.040		0.86	<0.001
δ*P*O_2_	−32.260 ± 3.792	0.030 ± 0.043		−0.01	0.495
*P*O_2_ (corrected for *P*CO_2_)	−18.203 ± 3.634	0.731 ± 0.030	0.543 ± 0.085	0.92	<0.001
sO_2_	−11.959 ± 7.230	1.014 ± 0.091		0.72	<0.001
δsO_2_	−34.715 ± 10.847	0.249 ± 0.136		0.046	0.074
*P*CO_2_	1.911 ± 0.327	0.912 ± 0.010		0.99	<0.001
δ*P*CO_2_	7.663 ± 1.401	−0.291 ± 0.044		0.45	<0.001
Hct	−1.508 ± 1.268	0.906 ± 0.033		0.94	<0.001
δHct	−16.752 ± 3.316	0.088 ± 0.087		0.00	0.318
[Hb]	−0.442 ± 0.232	1.209 ± 0.129		0.63	<0.001
δ[Hb]	−30.917 ± 12.805	14.967 ± 7.115		0.06	0.041
HemoCue [Hb]	−0.272 ± 0.211	1.408 ± 0.116		0.73	<0.001
HemoCue δ[Hb]	8.865 ± 11.673	9.215 ± 6.440		0.02	0.159

Linear regressions are according to: PCA(*x*) = *a* + *b* × control(*x*) and δ(*x*) = *a* + *b* × control(*x*). Multiple linear regression is according to: i-STAT *P*O_2_ = *a + b* × control *P*O_2_ + *c* × control *P*CO_2_. Abbreviations: [Hb], haemoglobin concentration; Hct, haematocrit; PCA, portable clinical analyser; *P*CO_2_, partial pressure of CO_2_; *P*O_2_, partial pressure of O_2_; and sO_2_, haemoglobin-O_2_ saturation.

### Partial pressure of O_2_

**Figure 2:**
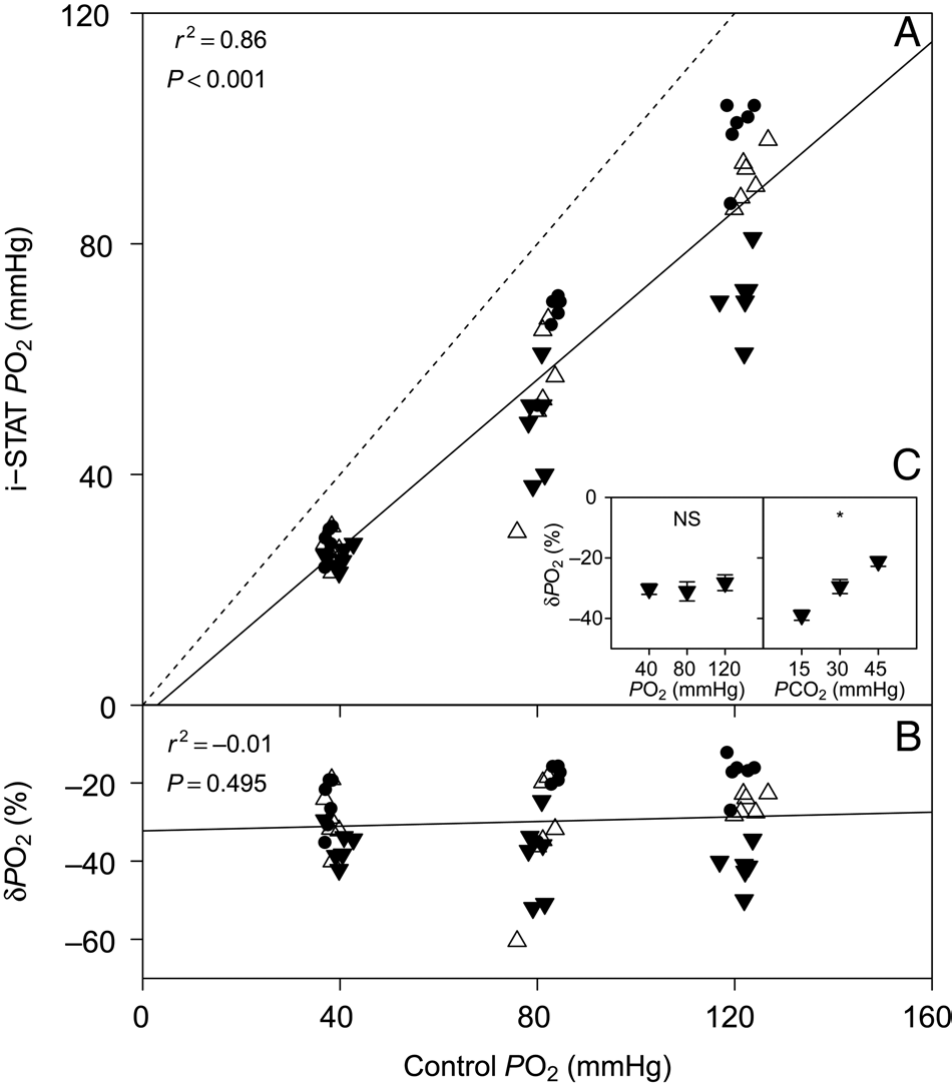
(**A**) Bar-headed goose whole blood *P*O_2_ measured with the i-STAT system vs. *P*O_2_ measured with a thermostated electrode (control) at 15 (inverted filled triangles), 30 (upright open triangles) and 45 mmHg *P*CO_2_ (filled circles). (**B**) The relative error of i-STAT *P*O_2_ measurements, δ*P*O_2_ vs. control *P*O_2_. (**C**) Effects of *P*O_2_ and *P*CO_2_ on δ*P*O_2_. See legend to Fig. 1 for further information.

We found a significant linear relationship between *P*O_2_ measured with the i-STAT and control *P*O_2_ measured with a thermostated electrode (Fig. 2A). However, no significant relationship was detected between δ*P*O_2_ and control *P*O_2_ (*P* > 0.05; Fig. 2B). The *P*CO_2_ had a significant effect on δ*P*O_2_ (*P* < 0.001), but no significant effect on δ*P*O_2_ was detected for *P*O_2_ (*P* = 0.723; Fig. 2C).

### Haemoglobin O_2_ saturation

**Figure 3:**
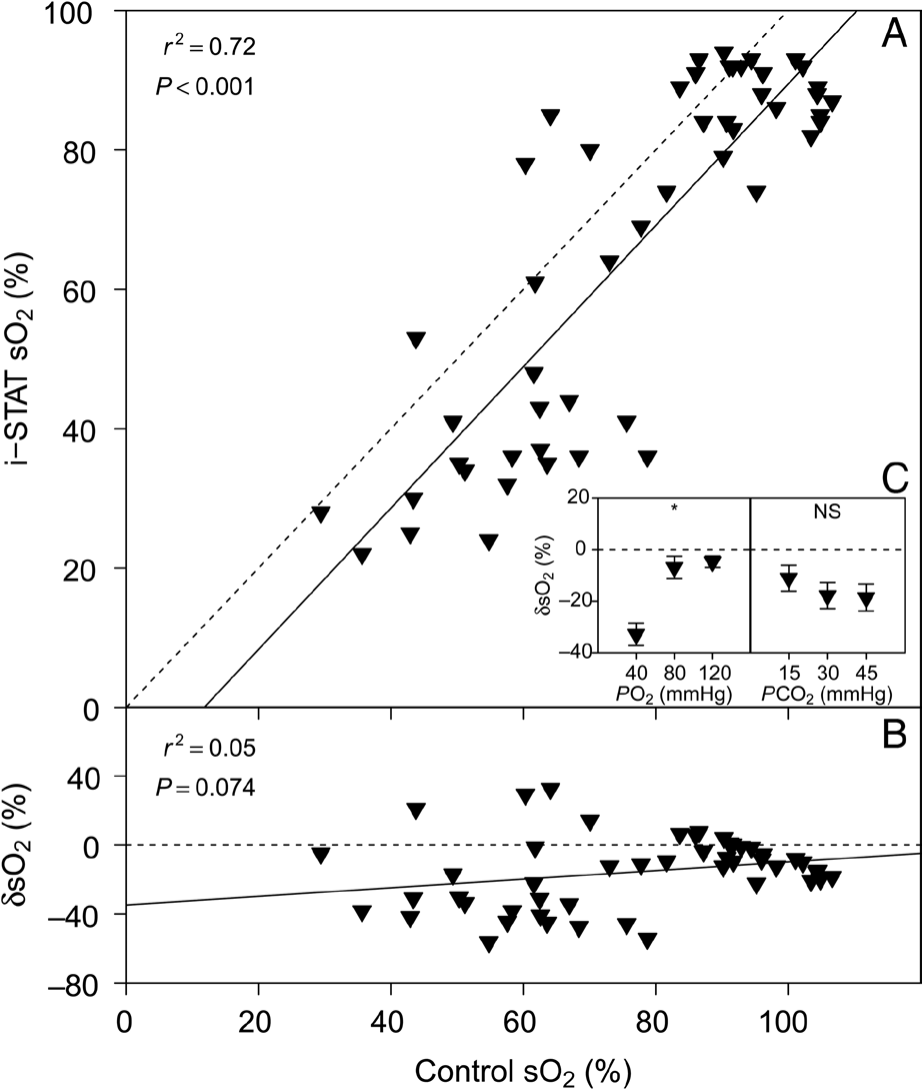
(**A**) Bar-headed goose haemoglobin-O_2_ saturation (sO_2_) measured with the i-STAT system vs. control sO_2_ measured according to Tucker (1967). (**B**) The relative error of i-STAT sO_2_ measurements, δsO_2_ vs. control sO_2_. (**C**) Effects of *P*O_2_ and *P*CO_2_ on δsO_2_. See legend to Fig. 1 for further information.

There was a significant linear relationship between sO_2_ measured with the i-STAT and control sO_2_ determined according to Tucker (1967; Fig. 3A), but we found no significant relationship between δsO_2_ and control sO_2_ (*P* > 0.05; Fig. 3B). A significant effect of *P*O_2_ on δsO_2_ was detected (*P* < 0.001), but not for *P*CO_2_ (*P* = 0.875; Fig. 3C).

### Partial pressure of CO_2_

**Figure 4:**
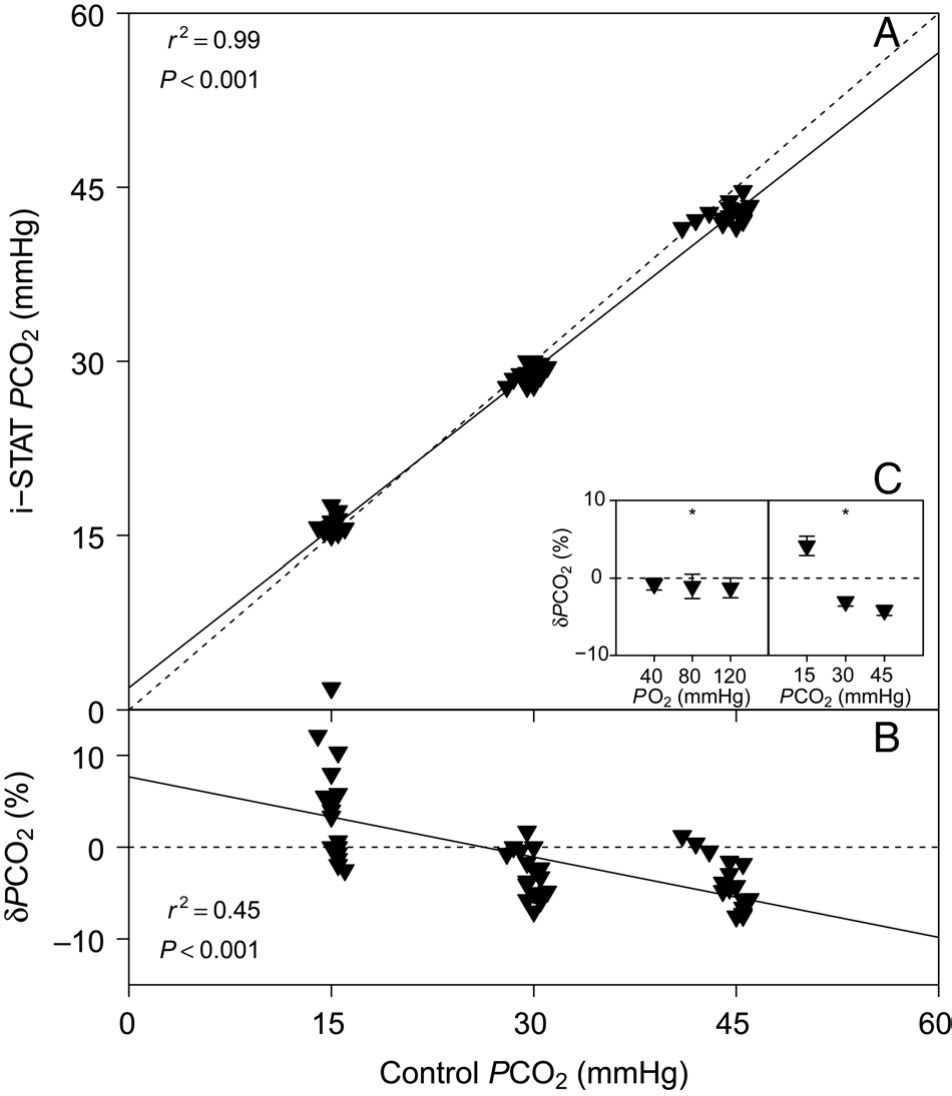
(**A**) Bar-headed goose whole blood *P*CO_2_ measured with the i-STAT system vs. *P*CO_2_ measured with a thermostated electrode (control). (**B**) The relative error of i-STAT *P*CO_2_ measurements, δ*P*CO_2_ vs. control *P*CO_2_. (**C**) Effects of *P*O_2_ and *P*CO_2_ on δ*P*CO_2_. See legend to Fig. 1 for further information.

There was a highly significant linear relationship between *P*CO_2_ measured with the i-STAT and control *P*CO_2_ measured with a thermostated electrode (Fig. 4A). Also, we detected a significant linear relationship between δ*P*CO_2_ and control *P*CO_2_ (Fig. 4B). Both *P*O_2_ (*P* = 0.047) and *P*CO_2_ (*P* = 0.017) had significant effects on δ*P*CO_2_ (Fig. 4C).

### Haematocrit

**Figure 5:**
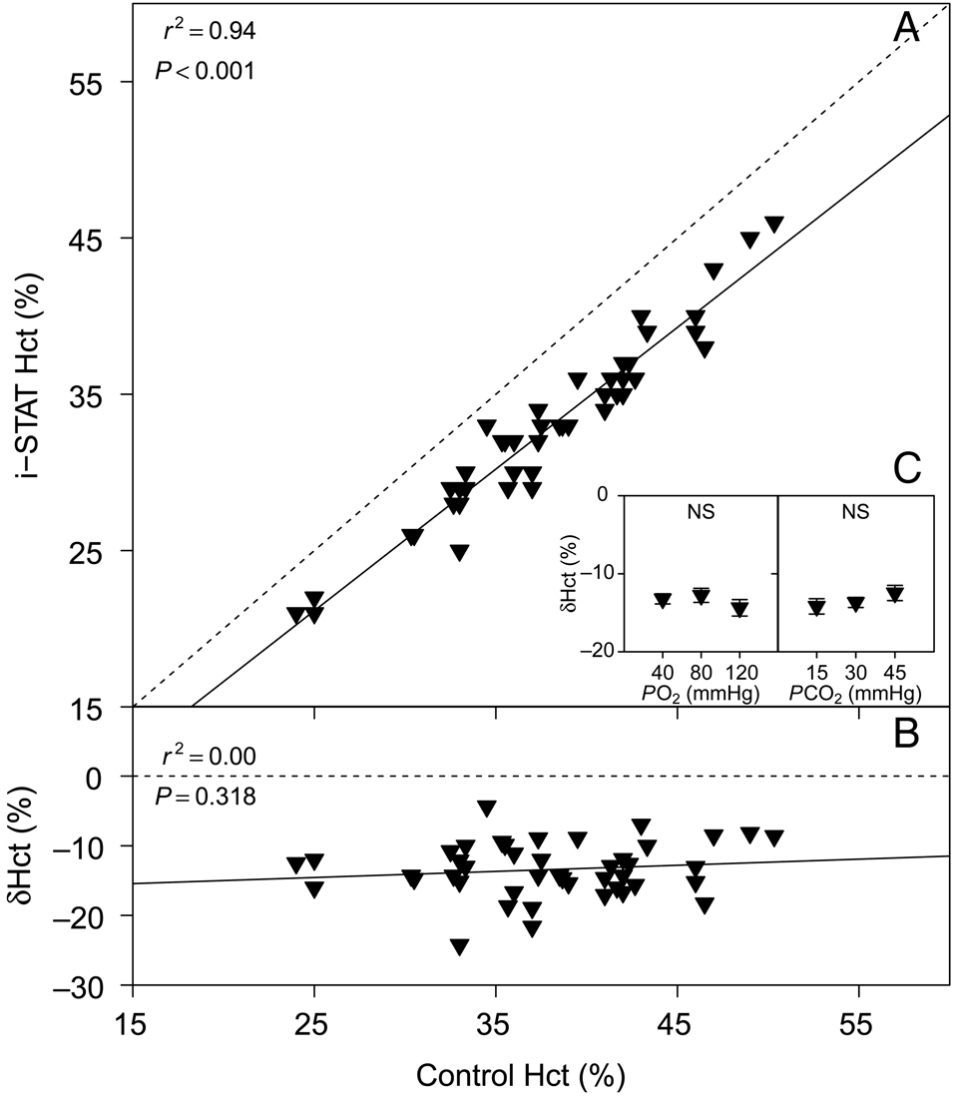
(**A**) Bar-headed goose haematocrit (Hct) measured with the i-STAT system vs. Hct measured in microcapillary tubes (control). (**B**) The relative error of i-STAT Hct measurements, δHct vs. control Hct. (**C**) Effects of *P*O_2_ and *P*CO_2_ on δHct. See legend to Fig. 1 for further information.

The average initial Hct for all sampled birds was 37.8 ± 0.8%, and no significant changes in Hct were detected throughout the tonometry trials (*P* > 0.05). We found a significant linear relationship between i-STAT Hct and control Hct measured in capillary tubes after centrifugation (Fig. 5A). There was, however, no significant relationship between δHct and control Hct (*P* > 0.05; Fig. 5B) and no significant effects of *P*O_2_ (*P* = 0.707) or *P*CO_2_ (*P* = 0.442) on δHct (Fig. 5C).

### Haemoglobin concentration

**Figure 6:**
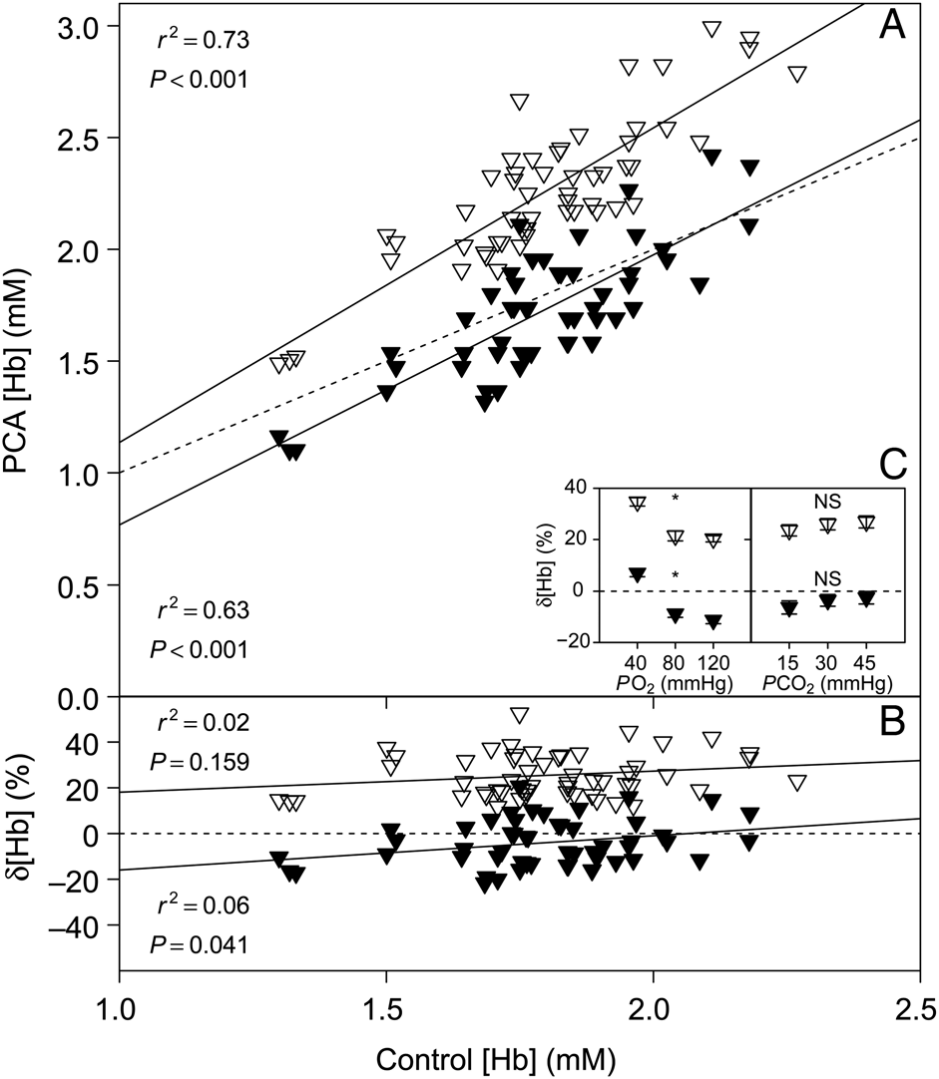
(**A**) Bar-headed goose haemoglobin concentration [Hb] measured with the i-STAT system (filled symbols) and the HemoCue (open symbols) vs. [Hb] measured with a spectrophotometer (control). (**B**) The relative error of i-STAT or HemoCue [Hb] measurements, δ[Hb] vs. control [Hb]. (**C**) Effects of *P*O_2_ and *P*CO_2_ on δ[Hb]. See legend to Fig. 1 for further information.

Average [Hb] throughout the study was 1.80 ± 0.03 mM. We found significant linear relationships between [Hb] measured with the i-STAT or the HemoCue and control [Hb] measurements with a spectrophotometer (Fig. 6A). In addition, we found a significant linear relationship between i-STAT δ[Hb] and control [Hb], but not between HemoCue δ[Hb] and control [Hb] (Fig. 6B). A significant effect of *P*O_2_ on δ[Hb] was detected for the i-STAT (*P* < 0.001) and the HemoCue (*P* < 0.001), but *P*CO_2_ had no significant effect on δ[Hb] in either the i-STAT (*P* = 0.507) or the HemoCue (*P* = 0.565; Fig. 6C).

## Discussion

Based on our results, we consider the i-STAT system a useful tool for measuring bar-headed goose whole blood pH, *P*O_2_, *P*CO_2_ and Hct; however, the i-STAT cannot produce reliable sO_2_ values, and the HemoCue system seems to provide more robust measurements of [Hb]. We typically found differences between i-STAT and control measurements, but in most cases both measurements scaled linearly and thus the equations presented in Table 1 can be used to correct PCA values. The critical researcher should, however, consider the limitations of the i-STAT system and their potential implications for the validity of the generated results, which are discussed in more detail in the following paragraphs. For more information on the methodology used by the i-STAT system, we refer to a summary published by Harter *et al*. (2014; Table 2) or to the i-STAT procedure manual for more detail (i-STAT Procedure Manual, 2014).

Our results indicate that pH measurements with the i-STAT system on bar-headed goose whole blood scaled linearly with control pH measurements performed with a thermostated electrode. The variation of i-STAT pH measurements was ∼0.1 pH units and, on average, the i-STAT underestimated control pH by ∼2%. These results are consistent with previous validation studies of the i-STAT system on fish (Harrenstien *et al.*, 2005; Gallagher *et al.*, 2010; Harter *et al.*, 2014, 2015); however, it remains unclear why the i-STAT underestimates blood pH across the different species that have been tested. Given the small measurement error of i-STAT pH measurements (δpH) in the bar-headed goose, it seems possible to correct these values with the linear equations reported in Table 1, if greater accuracy is required. The significant effects of *P*O_2_ and *P*CO_2_ on δpH indicate that changes in these factors will affect the accuracy of i-STAT pH measurements and therefore, it cannot be recommended to extrapolate these linear corrections beyond the tested conditions.

The *P*O_2_ measurements performed with the i-STAT displayed a large variability compared with control *P*O_2_ measurements with a thermostated electrode. This variability in *P*O_2_ measurements increased at higher *P*O_2_ tensions. As a consequence, δ*P*O_2_ was also highly variable and ranged from −10 to −50% at 120 mmHg *P*O_2_. This is in line with the results of a previous i-STAT validation study on sandbar shark (*Carcharhinus plumbeus*), which described increasing variability of *P*O_2_ measurements at higher *P*O_2_ tensions (Harter *et al.*, 2015). In the sandbar shark, this heterogeneity of variances was explained largely by differences in treatment temperature (tested at 15, 20 and 25°C) and the effects of a closed system temperature increase during measurements (Malte *et al.*, 2014). Given that the i-STAT system was developed for the analysis of human blood, samples in cartridges are heated to 37°C. However, the i-STAT does not have a cooling function, and if blood is loaded at 41°C (such as for the bar-headed goose), the small volume of just 90 μl is likely to cool, to some unknown degree (between 41 and 37°C), during analysis (∼2 min). However, we do not expect this relatively small temperature gradient (∼4°C) to have a major impact on blood *P*O_2_ due to the effects of closed system cooling.

The significant effect of *P*CO_2_ on δ*P*O_2_, however, indicates that changes in *P*CO_2_ affect the accuracy of i-STAT *P*O_2_ measurements and therefore may partly explain the observed variability. In fact, if i-STAT *P*O_2_ measurements are grouped by *P*CO_2_ (as in Fig. 2), a clear trend emerges, indicating that δ*P*O_2_ will be smallest at 45 mmHg *P*CO_2_ and will increase at lower *P*CO_2_ tensions (Fig. 2C). The arterial *P*CO_2_ of bar-headed goose blood has been shown to fall below 10 mmHg in hyperventilating birds in severe hypoxia (Scott and Milsom, 2007). Human arterial *P*CO_2_ (∼40 mmHg; Crosby and Robbins, 2003) closely matches our highest tested *P*CO_2_ (45 mmHg), and given that the i-STAT is optimized for the analysis of human blood, it is not surprising that highest accuracy of *P*O_2_ measurements is achieved in these conditions. To correct i-STAT *P*O_2_ measurements on bar-headed goose blood by taking into account both *P*O_2_ and *P*CO_2_, a multiple linear regression model was fitted to the data in Fig. 2A, and the resulting equation is presented in Table 1 (see *P*O_2_ corrected for *P*CO_2_). In fact, the addition of *P*CO_2_ as a predictor of i-STAT *P*O_2_ significantly increased *r*^2^ from 0.86 to 0.92 (One-way ANOVA, *P* < 0.001). The mechanism by which *P*CO_2_ affects the accuracy of i-STAT *P*O_2_ measurements, however, is not known. It seems possible that in fact it is changes in blood pH (that are intrinsically associated with changes in *P*CO_2_) and therefore changes in the redox potential of the sample that may affect the reading of the amperometric *P*O_2_ sensor within the i-STAT cartridge. This, however, remains to be tested thoroughly. As a result of these uncertainties, we emphasize that the linear equations presented here are likely to be applicable only to bar-headed goose whole blood under the tested conditions. Correction of i-STAT results should always be performed with caution, and additional validations for the specific study conditions are recommended.

Measurements of sO_2_ with the i-STAT system showed a significant linear relationship with control sO_2_ measurements made using the method of Tucker (1967). On average, the i-STAT underestimated control sO_2_ by ∼40% at low Hb-O_2_ saturations, but by <10% at full Hb saturation (Fig. 3B). The variability of sO_2_ measurements was large, however, and several replicate samples would be required to obtain a mean value that could be corrected into a more accurate measurement using our linear equations. A significant effect of *P*O_2_ on δsO_2_ indicates that changes in this factor will influence the accuracy of i-STAT sO_2_ measurements, and it seems that this will predominantly be the case at low *P*O_2_ tensions (Fig. 3C). The i-STAT system calculates sO_2_ from measured *P*O_2_ and pH and calculated HCO_3_^−^ values, by assuming a constant Hct and a ‘normal’ Hb-O_2_ affinity of human blood. Thus, this system cannot account for inter-specific differences in Hb-O_2_ affinity (or its modulation by allosteric effectors), fluctuations in Hct or the presence of dysfunctional Hb species (e.g. met-, sulf- and carboxyHb; i-STAT Technical Bulletin, 2013b). Therefore, we cannot recommend the i-STAT system for the analysis of sO_2_ in birds (including the bar-headed goose) or any other non-human species (Harter *et al.*, 2014, 2015) without thoroughly validating the results for the specific study conditions.

Measurements of blood *P*CO_2_ with the i-STAT system corresponded well with control measurements using a thermostated electrode. We detected a highly significant linear relationship between values generated with both methods, which accounts for 99% of the observed variation (*r*^2^ = 0.99; Fig. 4A). Over the range of tested *P*CO_2_, δ*P*CO_2_ was within 10% of control measurements. The accuracy of these results can be improved further by using the linear equation described in Table 1. However, extrapolation of results beyond the conditions that we tested is not recommended, owing to significant effects of *P*O_2_ and *P*CO_2_ on δ*P*CO_2_. Especially low *P*CO_2_ tensions will decrease the accuracy of i-STAT *P*CO_2_ measurements, and this result is consistent with previous studies that validated the i-STAT for the low blood *P*CO_2_ tensions observed in fish (Harter *et al.*, 2014, 2015). As previously discussed, we found that *P*O_2_ and *P*CO_2_ measurements in the i-STAT do influence one another, even though they are measured separately. Overall, we consider the i-STAT system a reliable tool to assess *P*CO_2_ in bar-headed goose whole blood, under the conditions that we tested. Users should bear in mind, however, that in severe hypoxia the arterial *P*CO_2_ of bar-headed goose blood can fall below 10 mmHg (Scott and Milsom, 2007), which may bode poorly for an accurate measurement of *P*CO_2_ (and *P*O_2_) with the i-STAT system.

Measurements of Hct performed with the i-STAT system were consistently lower than control measurements in capillary tubes, on average by ∼15% (Fig. 5B). However, there was a highly significant linear relationship between i-STAT Hct and control Hct, which accounted for 94% of the observed variation (*r*^2^ = 0.94). We found no significant effects of *P*O_2_, *P*CO_2_ or Hct on δHct (Fig. 5B and C); therefore, we can recommend correcting i-STAT Hct measurements on bar-headed goose blood with the linear equation provided in Table 1. The i-STAT system measures Hct by means of whole blood conductometry, where a higher fraction of RBCs will decrease sample conductivity. This measurement is corrected for temperature, sample volume and plasma ion levels (albeit only Na^+^ and K^+^). In fish, the i-STAT generally underestimated Hct by 30–45% (Harrenstien *et al.*, 2005; DiMaggio *et al.*, 2010; Harter *et al.*, 2014, 2015), suggesting that the conductive properties of a whole blood sample from bar-headed geese resembles the characteristics of human blood more closely than that of fish. However, in all of the above studies, Hct was underestimated by the i-STAT, and this may be a consequence of the nucleated state of RBCs in both fish and birds. Mammals (including humans) have non-nucleated RBCs. Therefore, researchers using the i-STAT system on any non-mammalian species can expect an underestimation of Hct and need to validate these results appropriately.

We performed simultaneous measurements of [Hb] with two commonly used PCAs, the i-STAT and the HemoCue, and compared these values with [Hb] measured with a spectrophotometer using the cyanomethaemoglobin method. Values generated with both PCAs yielded significant linear relationships with control measurements (Fig. 6A); however, δ[Hb] was smaller in the i-STAT compared with the HemoCue, which overestimated control [Hb] by ∼20% (Fig. 6B). While the average [Hb] measured by the i-STAT was consistent with control measurements, there was a substantial amount of variation, which may require increasing the number of replicate samples to obtain an accurate mean [Hb]; the same applies for the HemoCue. Considering that the i-STAT does not measure [Hb], but calculates it from Hct (simply by multiplying values by 0.34; i-STAT Technical Bulletin, 2013a), it is surprising that i-STAT measurements were more accurate than those obtained from the HemoCue, which measures [Hb] photometrically after conversion to azide-methaemoglobin (HemoCue Manual, 2015). Given that in the i-STAT, Hct was underestimated by ∼15% and [Hb] was not, it seems that the high accuracy of [Hb] measurements was rather coincidental, which could be verified using broader ranges of Hct and [Hb] than those used here. The simple algorithm used by the i-STAT to convert Hct measurements into [Hb] values is highly susceptible to changes in mean corpuscular [Hb], which may occur with changes in the physiological status of the bird (e.g. stress, exercise or osmotic disturbances; Riddick *et al.*, 1971; Nikinmaa and Huestis, 1984; Prats *et al.*, 1996) or changes in environmental conditions (e.g. hypoxia; Black and Tenney, 1980). Given that the HemoCue genuinely measures [Hb], the values obtained are likely to be more robust to inter-specific differences and other confounding factors associated with the physiological status of the animal or environmental conditions. Therefore, and despite the lower accuracy compared with the i-STAT system, we consider the HemoCue the better instrument for measuring [Hb] in bar-headed goose whole blood. The highly significant linear relationship between HemoCue [Hb] and control values (Fig. 6A) indicates that a correction of HemoCue results is possible with the linear equation provided in Table 1. However, changes in *P*O_2_ (Fig. 6C) may affect the accuracy of HemoCue (and i-STAT) [Hb] measurements and likewise the validity of the correction equation. We also emphasize that the obtained results may vary with species, even among birds.

### Conclusion

The i-STAT system is a reliable tool for measuring blood parameters in the bar-headed goose. For whole blood pH, *P*O_2_, *P*CO_2_ and Hct, we found significant differences between i-STAT and control measurements; however, in general these results can be corrected by using the linear equations provided in Table 1, thereby allowing researchers to increase the accuracy of i-STAT results. The sO_2_ measured with the i-STAT displayed a substantial variability and is likely not a robust measurement over a broader range of study conditions or species. While the i-STAT system yielded satisfactory measurements for [Hb] in bar-headed goose whole blood, we consider the HemoCue a more reliable tool to assess this blood parameter, as long as the results are corrected. We emphasize that the linear equations presented here are valid only for the range of conditions that we tested and that extrapolation beyond these values or their application to other species (including other birds) will require appropriate validation.

## Funding

This study was supported by Natural Sciences and Engineering Research Council (NSERC) of Canada Discovery Grants to C.J.B. and W.K.M. and an NSERC Accelerator Supplement to C.J.B.
